# Piloting wastewater-based monitoring on a university campus to inform public health surveillance and response for opioids and other high-risk substances

**DOI:** 10.2166/wh.2026.182

**Published:** 2026-01-12

**Authors:** Daniel Gerrity, Casey A. Barber, Rebecca A. Trenholm, Andrew Black, Edwin C. Oh, Anil T. Mangla, Cassius Lockett, Brett J. Vanderford

**Affiliations:** a Southern Nevada Water Authority, Las Vegas, NV 89193, USA; b Department of Civil & Environmental Engineering, University of Wisconsin-Platteville, Platteville, WI, USA; c Laboratory of Neurogenetics and Precision Medicine, College of Sciences, University of Nevada, Las Vegas, NV, USA; d Neuroscience Interdisciplinary Ph.D. Program, Kirk Kerkorian School of Medicine, University of Nevada, Las Vegas, NV, USA; e Department of Brain Health, Kirk Kerkorian School of Medicine, University of Nevada, Las Vegas, NV, USA; f Department of Internal Medicine, Kirk Kerkorian School of Medicine, University of Nevada, Las Vegas, NV, USA; g Southern Nevada Health District, Las Vegas, NV, USA; h School of Public Health, University of Nevada, Las Vegas, NV, USA

**Keywords:** fentanyl, illicit drugs, overdose, surveillance, wastewater-based epidemiology, wastewater surveillance

## Abstract

This study demonstrates the use of wastewater monitoring for opioids and other high-risk substances on a university campus, intended to inform public health surveillance and response efforts for youth and transitional age youth. Over a 14-week campaign, we collected 54 grab samples from three campus manholes, including one that isolated flows from a student housing complex, and analyzed them for 24 parent compounds and metabolites using LC-MS/MS. Heroin and its metabolite 6-acetylmorphine were among several opioid-related detections, illustrating how campus wastewater monitoring can capture actionable use events for targeted public health interventions. Fentanyl, norfentanyl, and xylazine were consistently below their method reporting limits across all 54 samples, consistent with recent declines in national overdose and wastewater datasets. The highest detection frequencies were associated with methamphetamine (22–50%), amphetamine (39–83%), and a THC metabolite (50–100%). This study also highlights the implications of sample type (grab vs. composite) and day of week for wastewater-based epidemiology (WBE). Considering that the use of opioid-related WBE as an actionable public health surveillance tool is still relatively new, additional case studies are needed to explore the potential of this emerging tool and increase confidence in deploying public health interventions in response to wastewater data.

## Graphical Abstract

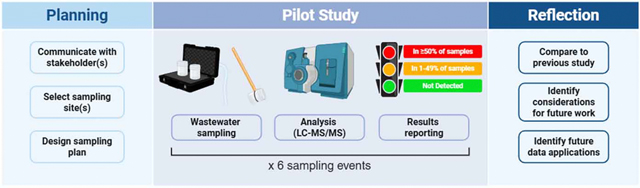

## 1. INTRODUCTION

Data for 2022–2023 from the Centers for Disease Control and Prevention (CDC) indicate that the age-adjusted fatal overdose rate in the United States (U.S.) fell for the first time in 20 years (Garnett & Miniño 2024), presumably due to the ongoing public health response to the opioid epidemic (HHS 2018). Although there are indications this promising trend may continue, overdoses remain one of the leading causes of death in the U.S. (CDC 2023; CDC Newsroom 2025). The opioid crisis has been attributed to multiple factors, including inappropriate prescribing practices and misuse of prescription opioids that led to a steady increase in fatal overdoses in the early 2000s (Compton et al. 2016). Around 2010, there was a rapid rise in heroin-associated overdoses, for reasons that are still under debate (Compton et al. 2016; Wolff et al. 2020), and this was followed in close succession by the emergence of increasingly potent synthetic opioids, namely fentanyl.

Despite improvements across the broader U.S., the COVID-19 pandemic exacerbated opioid-related health outcomes in several states (Garnett & Miniño 2024), including Nevada, where the statewide opioid overdose mortality rate more than doubled between 2019 and 2023 (SNHD 2024). Based on provisional data for 2024, Nevada and South Dakota were the only two U.S. states that continued to see slight increases in drug overdose deaths (CDC National Center for Health Statistics 2025). In 2023 alone, there were 6,401 total overdoses (ODMAP 2025) and 691 overdose deaths (SNHD Vital Records Data) in Clark County, which includes the Las Vegas metropolitan area in Southern Nevada. In 2023, among Clark County’s youth (0–18) and transitional age youth (TAY; 18–24), hospital discharge data indicate that 139 individuals were seen in emergency rooms for opioid-related reasons. During the same year, 25 opioid-related deaths among Clark County residents aged 0–24 were recorded.

Existing local, state, and national data sources may underestimate the true occurrence of drug use behaviors, opioid use disorder (Bharel 2019), and drug-related health outcomes, which, like infectious diseases, can be subject to underreporting and under-ascertainment (Gibbons et al. 2014). Public health surveillance approaches to this issue have known limitations; for example, self-reporting of stigmatized behaviors may be inaccurate (Steinhoff et al. 2023). Other approaches involve indirect indicators of consumption (e.g., prescribing data, drug checking, posts on online forums), post-overdose healthcare utilization (e.g., emergency medical services calls, emergency department encounters, or inpatient admissions), and vital records reporting of overdose deaths. Each of these data sources offers valuable but incomplete information; changes in prescribing data, for example, do not fully explain increases in synthetic opioid-related deaths (Olfson et al. 2023). Timeliness is another concern, as overdose death reporting can take up to several months before actionable data become available (Fliss et al. 2021).

Early in the COVID-19 pandemic, Bivins et al. (2020) highlighted the value of wastewater monitoring as a complementary approach to public health surveillance of infectious disease, but the value of wastewater-based epidemiology (WBE) is not limited to pathogens. Even prior to the pandemic, researchers proposed applications of WBE for markers of chronic disease, chemical exposures, substance use, and more (Choi et al. 2018). Building on pandemic-related momentum and investments in wastewater monitoring, WBE could be further expanded to address public health surveillance data gaps for opioids and other high-risk substances (HRS) (Huizer et al. 2021).

Similar to applications elsewhere in the U.S. (Gushgari et al. 2019), Gerrity et al. (2024) used WBE to estimate HRS consumption in Southern Nevada and to identify spatial and temporal patterns in drug use, including a notable spike in fentanyl consumption in October 2022. Zhuang et al. (2024) then used this community-scale dataset to correlate HRS consumption with corresponding socioeconomic and demographic indicators. However, Mehrotra et al. (2023) noted that HRS monitoring at wastewater treatment facilities might not provide sufficient resolution for effective public health action, thereby highlighting the importance of sampling in upstream collection systems. For example, WBE efforts at schools/universities have demonstrated promise for targeted public health responses, including for COVID-19 (Betancourt et al. 2021; Harris-Lovett et al. 2021; Vo et al. 2022). Wastewater monitoring has also been used to assess drug use at primary, secondary, and/or post-secondary schools in Europe (Zuccato et al. 2017; Verovšek et al. 2021) and multiple locations within the U.S. (Heuett et al. 2015; Gushgari et al. 2018; NMDOH 2023). In fact, this approach has been used to assess drug consumption on university campuses during high-stress periods (e.g., finals week) (Burgard et al. 2013) and sporting events (Montgomery et al. 2021).

In Nevada, there is an urgent need for complementary public health surveillance approaches capable of generating accurate assessments of near-real-time conditions to inform youth overdose prevention and response activities. Building data collection capacity and supporting youth prevention programs are among the funding priorities for opioid settlement funds identified by the Johns Hopkins Bloomberg School of Public Health (JHBSPH 2025); over $1.2 billion of these funds have been allocated to support Nevada’s response to the opioid epidemic as of 2025 (Hill 2025). Consistent with the JHBSPH guiding principles for this funding, this study set out to respond to the overdose crisis among youth and TAY (Friedman & Hadland 2024) by filling a critical data gap related to drug use on a college campus (Noguchi 2024) and to compare findings to those of the surrounding sewershed in the prior Gerrity et al. (2024) study. Using this approach as a model for broader implementation, future applications could prioritize deployment of targeted public health actions, such as awareness campaigns and naloxone distribution and training, to help reduce drug use and overdoses among youth and TAY (Viloria 2002).

## 2. METHODS

### 2.1. Study site

**Figure 1 |:**
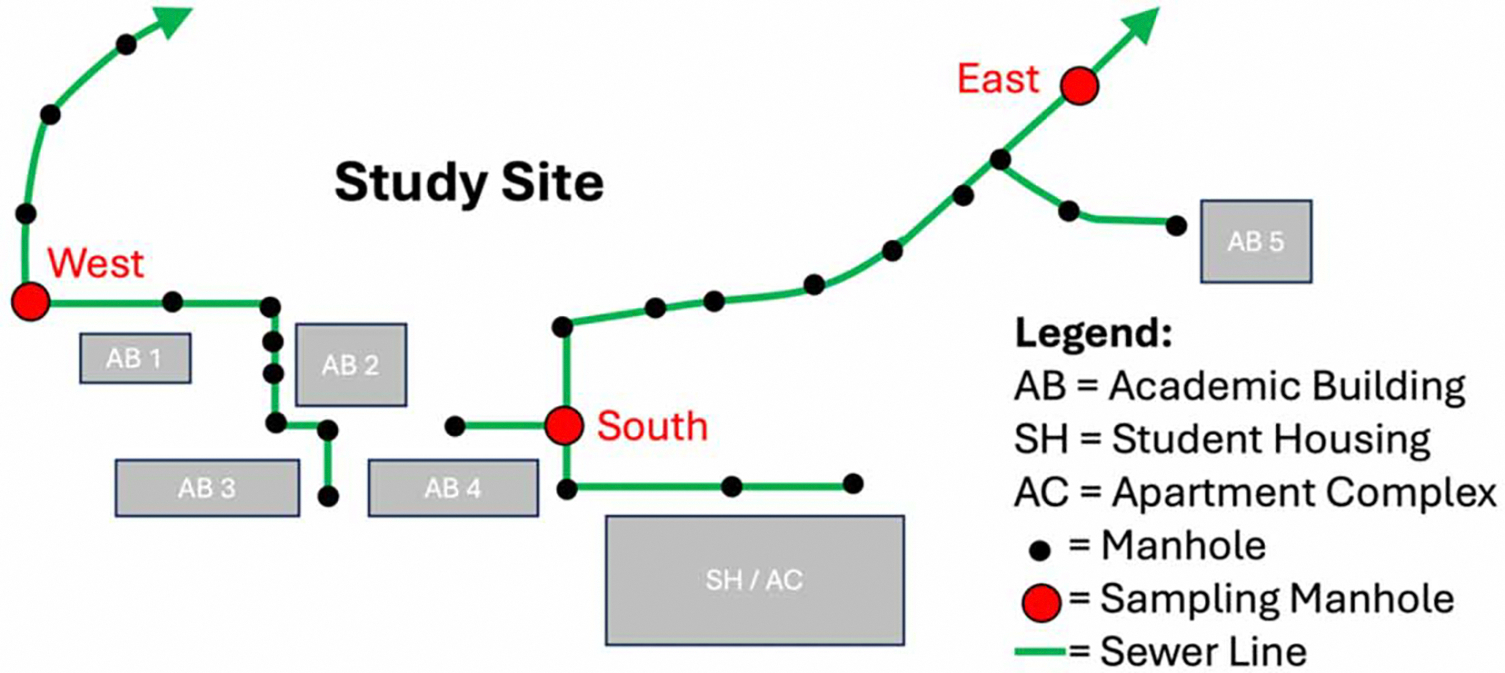
Map of the university campus and associated sewer infrastructure. See Figure S1 for a photo of the South manhole illustrating how student housing complex flows were isolated.

This pilot wastewater monitoring program was conducted during the spring/summer of 2025 on a university campus located in Southern Nevada, USA. During the spring 2025 semester, on-campus enrollment was approximately 3,750 students, with >98% at the undergraduate level. The campus includes five academic buildings and an approximately 350-bed, 8-building university-associated housing complex for both students and non-students (Figure 1).

### 2.2. Sample collection

Prior to initiating the sampling campaign, the project team contacted the local municipality (i.e., wastewater utility) and the university to explain the objectives of the project and to seek approval to sample. The project team then collaborated with these stakeholders to identify utility access points (i.e., manholes) that would allow for isolation of sewage from certain buildings, thereby facilitating future public health interventions (if warranted). This resulted in the selection of three manholes representing the West, South, and East portions of campus (Figure 1). The West manhole served three academic buildings, the South manhole allowed for isolation of flows from the housing complex (see Figure S1  in the Supplementary Information file), and the East manhole represented a downstream blend of two academic buildings and the housing complex. Regarding privacy protection, it was not possible to trace wastewater samples to specific individuals or rooms within the contributing buildings.

**Figure 2 |:**
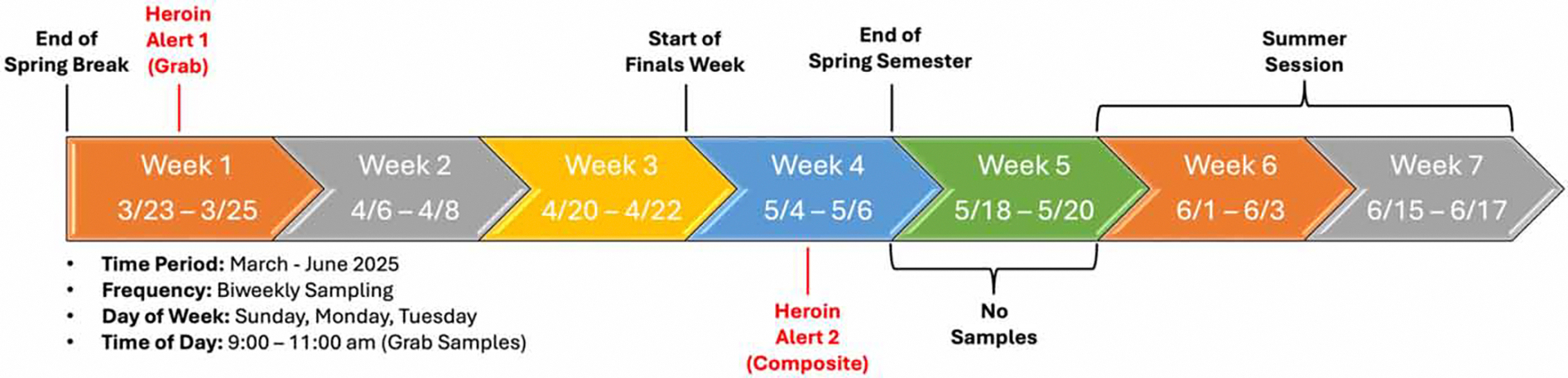
Timeline of the university campus wastewater sampling campaign.

Sampling was planned for every two weeks over a 14-week period spanning from late March through mid-June 2025 (Figure 2); samples were not collected during the fifth sampling week to allow for monitoring of an unrelated, off-site special event (data not included in this study). The remaining six sampling weeks included the end of spring break through the end of the spring semester (i.e., finals week) and a portion of the summer session.

Because automated composite samplers were not available at the start of the study, each sampling week consisted of individual grab samples collected from each manhole with a telescoping pole at ~10:00 am on Sunday, Monday, and Tuesday mornings (*n* = 18 samples per manhole; 54 samples total). On a single occasion during the fourth sampling week, a composite sample was collected from the South manhole to allow for a direct comparison of grab vs. composite sampling. That sample was collected using an automated composite sampler suspended within the manhole (CA101, C.E.C. Analytics, Calgary, AB, Canada). The sampler was programmed to collect a single aliquot (~30 mL) each hour for 24 hours between 10:00 am Sunday morning (5/4/2025) through 10:00 am Monday morning (5/5/2025). More details related to the autosampler and its configuration are provided in Figure S2.

Samples were collected in new high-density polyethylene (grab) or polypropylene (composite) bottles and then immediately transferred to new 40-mL amber glass vials containing 50 mg/L of ascorbic acid for oxidant quenching and 1 g/L of sodium azide for biological preservation. Oxidants were not expected in any samples, but inclusion of ascorbic acid is standard practice for the laboratory in this study. Note that an earlier HRS hold time study described potential instability of spiked heroin in the presence of ascorbic acid, although the original heroin signal could still be detected as the parent compound (within 7 days of sample collection) or as its major metabolite acetylmorphine (Gerrity et al. 2024). The amber glass vials were then transported to the laboratory and stored at 4 °C until processing and analysis. Weekly samples were processed and analyzed as a single batch, with the full sample set available on Tuesday afternoons and processing/analysis typically commencing on Wednesday mornings. Each sampling week also included one field blank (*n* = 6 total field blanks) consisting of Milli-Q water that was transferred to a sample vial at one of the manhole sampling sites; these samples were then processed in the same manner as actual samples. All field and analytical blanks were negative during the study.

### 2.3. Sample analysis

The suite of target analytes included 24 parent compounds and metabolites (Table S1). Target non-opioids included cocaine [also benzoylecgonine (BZE), ecgonine methyl ester (EME), ecgonine, and norcocaine], methamphetamine [also amphetamine], 3,4-methylenedioxy methamphetamine (MDMA) [also 3,4-methylenedioxyamphetamine (MDA)], delta-9-tetrahydrocannabinol (THC) [also 11-hydroxy-delta-9-tetrahydrocannabinol (THC-OH) and 11-nor-9-carboxy-delta-9-tetrahydrocannabinol (THC-COOH)], and the tranquilizer xylazine. Target opioids included natural [heroin, morphine, and codeine], semi-synthetic [hydrocodone and oxycodone], and synthetic [fentanyl, methadone, and tramadol] forms. Relevant opioid metabolites included acetylmorphine for heroin, norfentanyl for fentanyl, and 2-ethylidene-1,5-dimethyl-3,3-diphenylpyrrolidine (EDDP) for methadone. All standards and isotopes were purchased in methanol from Cerilliant (Round Rock, TX, USA), except for ecgonine which was purchased from Restek (Centre County, PA, USA).

Interpretation of some of these target compounds can be confounded by reactivity with quenching agents/preservatives (e.g., heroin to acetylmorphine) and aqueous instability (e.g., cocaine and its metabolites). Target compound stability during sewer system transport and laboratory storage were described in detail in Gerrity et al. (2024). Also, one or more of the major metabolites included in this study can also serve as parent compounds. This is best exemplified by the complicated interplay between heroin, acetylmorphine, morphine, codeine, and hydrocodone (Figure S3). Similarly, amphetamine is a metabolite of methamphetamine, but it can also be taken directly (e.g., Adderall) for treatment of attention-deficit hyperactivity disorder (ADHD).

The target compounds were analyzed with direct injection of aqueous samples after 10-fold dilution to reduce matrix interference; samples were diluted further if concentrations fell outside the calibration range. All compounds were analyzed by liquid chromatography tandem mass spectrometry (LC-MS/MS) with isotope dilution according to previously published methods (Gerrity et al. 2022b, 2024). Analysis employed a CTC Autosampler (CTC Analytics, Zwingen, Switzerland) and an Agilent 1260 LC Binary Pump (Palo Alto, CA, USA). Target compounds and their isotopically labeled analogs were then quantified with positive electrospray ionization (ESI) in multiple reaction monitoring (MRM) mode on a SCIEX 6500 QTRAP mass spectrometer (Redwood City, CA, USA; Table S2). Additional details for all target compounds, including method reporting limits (MRLs), calibration ranges, and MRM transitions, are included in Text S1.

## 3. RESULTS AND DISCUSSION

### 3.1. Project communication

An evaluation of college/university campus wastewater surveillance programs during the COVID-19 pandemic highlighted the importance of communication with diverse stakeholders for project success (Harris-Lovett et al. 2021). While the public is highly supportive of wastewater surveillance for COVID-19 and other infectious diseases, support wanes somewhat for more sensitive targets such as opioids and HRS (Holm et al. 2022, 2024), particularly when the effort is focused on youth [e.g., New Mexico’s high school program (NMDOH 2023)]. Thus, this project focused on proactively communicating its goals and addressing potential ethical concerns (Bowes et al. 2023). Figure S4 includes a flyer distributed to project stakeholders to describe key elements of the project, raise awareness of substance use in Nevada, explain data sharing and potential data uses, and highlight available resources related to addiction and recovery.

Results were shared electronically with project stakeholders as soon as the data were finalized, typically by the end of the sampling week or at the beginning of the following week when repeat analysis was needed (e.g., dilutions due to unexpectedly high concentrations of certain target compounds). Recipients included (1) the local health department, (2) the city’s mobile crisis intervention team, (3) the university student wellness team, (4) university administration, and (5) the collaborating wastewater utility.

### 3.2. High-risk substance detection frequencies and concentrations

**Figure 3 |:**
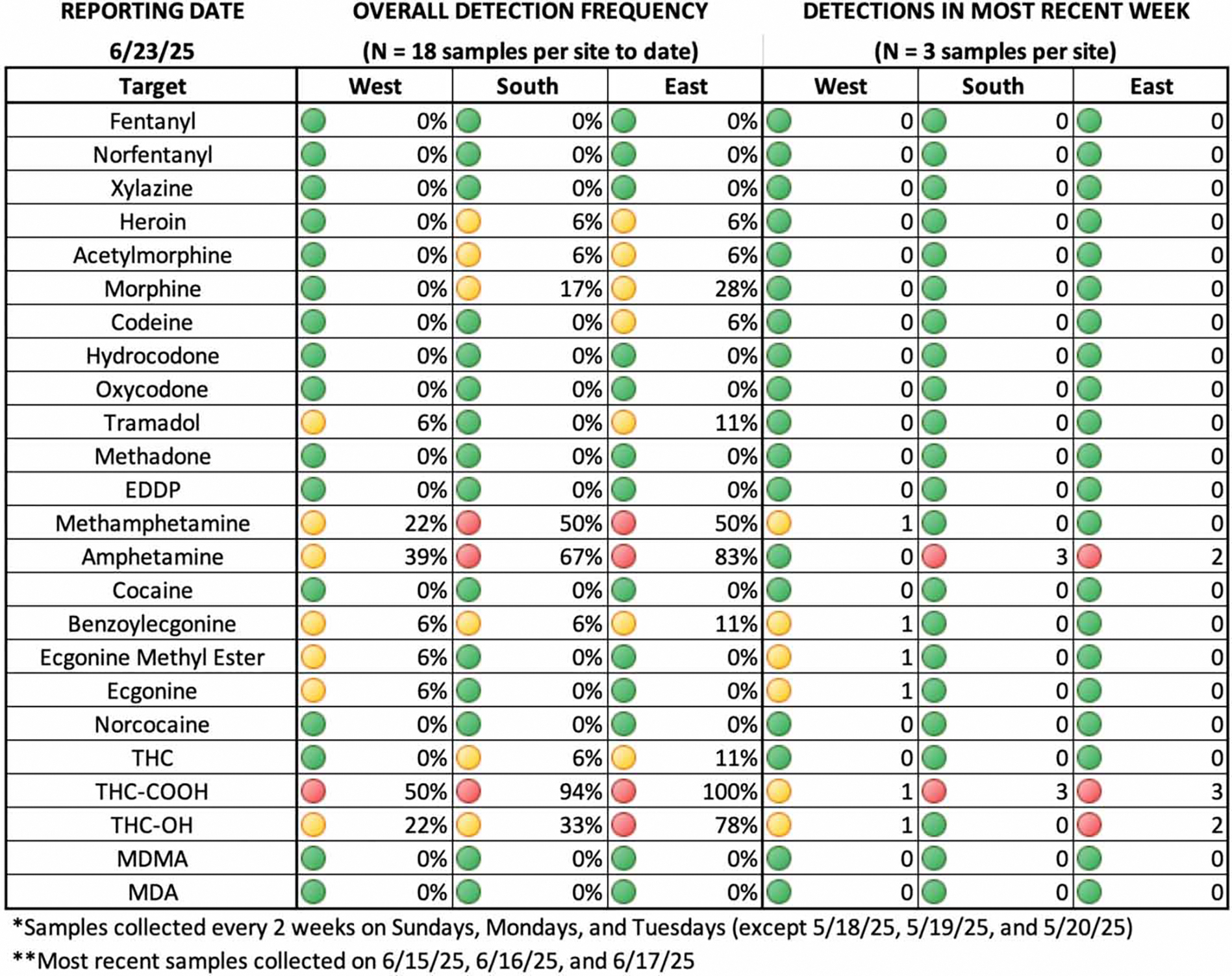
Example data summary submitted to project stakeholders. This data summary includes overall detection frequencies for each manhole sampling location across all sampling weeks, in addition to the number of detections at each manhole sampling location in week 7. Color scheme of circles was arbitrarily set for visualization purposes only (green = 0% detection frequency, 0 detections in current week; yellow= <50% detection frequency, 1 detection in current week; red = ≥50% detection frequency, ≥2 detections in most recent week).

**Figure 4 |:**
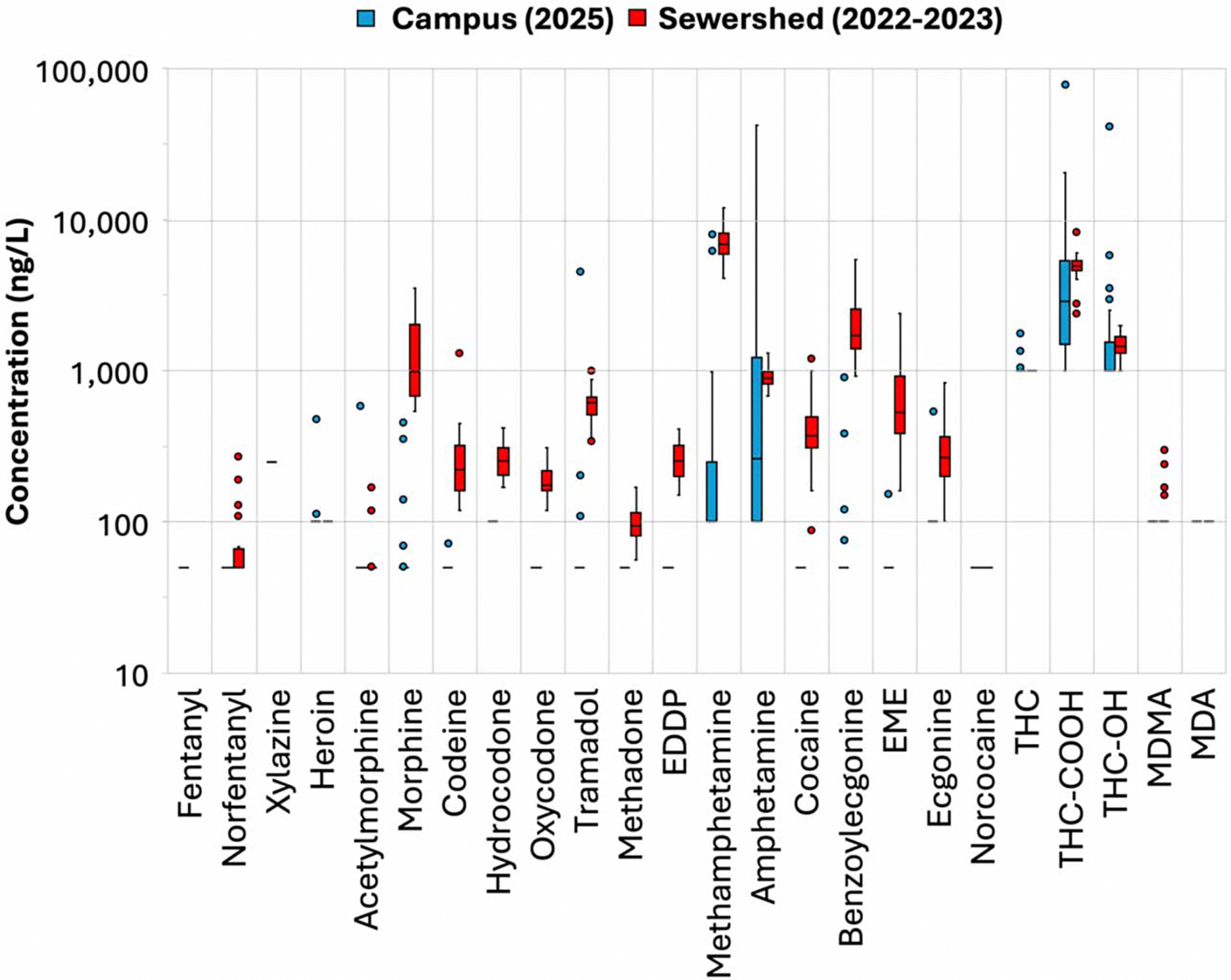
Box-and-whisker plots of target compound concentrations (ng/L) for the university campus (current study) and the corresponding sewershed [Facility 4B in Gerrity et al. (2024)]. Raw data for the university campus are available in Table S3. Campus plots represent data from 54 manhole samples (aggregate of all West, South, and East samples), and sewershed plots represent 26 biweekly wastewater treatment plant samples collected in 2022/2023. Plots are presented in the Tukey style, with boxes indicating the 25th, 50th (median), and 75th percentiles; whiskers denoting ± 1.5 times the interquartile range (IQR); and circles representing outliers based on the IQR method. The method reporting limit (MRL) was imputed for all samples with concentrations < MRL. Gerrity et al. (2024) did not monitor for fentanyl or xylazine.

Overall detection frequencies across all sampling weeks are summarized in Figure 3, which also represents the format in which the data were presented to project stakeholders. Each target compound was described in terms of overall detection frequency and the number of positive samples (i.e., >MRL) in the most recent sampling week. Statistical distributions for the target compound concentrations are illustrated in Figure 4, along with a comparison for the corresponding sewershed (Sewershed 4B) from 2022 to 2023 (Gerrity et al. 2024). Finally, all raw data are provided in Table S3. For clarity, we define a ‘detection’ as a concentration greater than the MRL; non-detects were imputed at the MRL for plotting in Figure 4.

#### 3.2.1. Priority targets for overdose prevention: fentanyl, norfentanyl, xylazine

During the previous 2022–2023 HRS wastewater surveillance study in Southern Nevada (Gerrity et al. 2024), the annual overdose rates (fatal + nonfatal across all drugs) were 66 and 141 per 100,000 people for the study site’s corresponding ZIP code and sewershed, respectively (ODMAP 2025). The sewershed-level overdose rate is partially explained by an adjacent ZIP code, which had an overdose rate of 216 per 100,000 people. Even in 2024, that adjacent ZIP code had one of the highest opioid-involved overdose death rates in Southern Nevada, at 35 per 100,000 people (SNHD 2024).

Nonetheless, in the current study, there were zero campus-based wastewater samples containing fentanyl, norfentanyl, or xylazine above the MRL across the entire sampling campaign, which is reassuring in terms of overdose prevention. Although fentanyl and xylazine were not included in the 2022–2023 study, norfentanyl was detected in 38% of 26 biweekly samples from the corresponding sewershed, including several of the study’s highest sucralose-normalized norfentanyl concentrations. Thus, there is precedent for detecting the major marker of fentanyl consumption in wastewater collected from the surrounding area. Also, there is evidence that overdose rates are highest in the late spring and early summer in Clark County (SNHD 2024), which aligns with the time period of the current study. Although the lack of fentanyl, norfentanyl, and xylazine may be specific to this particular campus, there is also supporting evidence from a national wastewater monitoring program that fentanyl consumption in Clark County had already started declining in the summer of 2024 (Figure S5) (Biobot 2023).

#### 3.2.2. Heroin and related targets: acetylmorphine and morphine

In the previous HRS wastewater surveillance study, heroin was never detected above the MRL in any of the 208 samples collected across 8 different sewersheds (Gerrity et al. 2024). This was attributed to several factors, including heroin’s potentially low concentrations overall, its slightly higher MRL (100 ng/L), and its instability in the presence of the quenching agent ascorbic acid. Instead, heroin consumption was assessed through metabolites (Figure S3), specifically via morphine concentrations after first differentiating morphine derived from heroin/acetylmorphine metabolism vs. direct morphine consumption. Although acetylmorphine is a more direct indicator of heroin consumption, morphine is more abundant and more reliable when developing consumption estimates (Gracia-Lor et al. 2016), at least for community-scale WBE. In Gerrity et al. (2024), acetylmorphine was detected in only 19% of samples from the sewershed in question, while morphine was detected in 100% of samples.

In the current study, acetylmorphine was detected in only two grab samples (Figure 3), both on the same date from the hydraulically linked South and East manholes (Table S3), while morphine was detected in 17 and 28% of samples from the South and East manholes, respectively. There was a second instance of acetylmorphine detection at the South manhole, and this is discussed later in relation to grab vs. composite sampling. Surprisingly, *heroin* was simultaneously detected with acetylmorphine in the same two grab samples from the South and East manholes. Compared to the 0% detection frequency in Gerrity et al. (2024), these heroin detections may have been driven by reduced dilution (i.e., no commercial/industrial flows), shorter travel time in the sewer collection system, and rapid sample turnaround time (~24 hours for these samples).

Although morphine might be appropriate for developing consumption estimates across larger services areas (i.e., sewersheds), the difficulty in distinguishing morphine from heroin metabolism vs. direct consumption adds uncertainty for collection system applications, particularly when considering deployment of public health interventions. In this case, these data are potentially actionable given the following: (1) the simultaneous detection of heroin and acetylmorphine in a given sample, (2) the consistency in hydraulically linked locations on the same date (3/25/25), and (3) the ability to link the signal to a specific location (i.e., the housing complex via the South manhole). The acetylmorphine concentrations in these samples (588 ng/L and 497 ng/L) were higher than all earlier detections in Gerrity et al. (2024), including for the sewershed in question (Figure 4), although differences in dilution are expected for upstream collection systems versus community-scale wastewater treatment facilities. Dumping of heroin cannot be ruled out in this instance, as post-disposal degradation of heroin could potentially lead to acetylmorphine detection (Gerrity et al. 2024). However, a public health response might still be warranted given the presence of heroin and the repeat detection (discussed later), although the type of response might be different than for a confirmed overdose.

#### 3.2.3. Other opioids: hydrocodone, oxycodone, methadone, EDDP, tramadol

Methadone and its major metabolite EDDP, hydrocodone, and oxycodone were <MRL in all samples in the current study. In the previous study (Gerrity et al. 2024), EDDP, hydrocodone, and oxycodone were detected in 100% of samples, while methadone was <MRL in certain sewersheds. This suggests that detection of certain opioids – including ones related to treatment – may be more impacted by sampling location than other targets. Particularly for hydrocodone and oxycodone, it is unclear why all samples were <MRL in the current study, so it can only be assumed that levels of consumption were too low to be detected with the given methods. However, tramadol was detected in three different samples, including a Tuesday sample from the West manhole and two different Monday samples from the East manhole. The week 2 detection of Tramadol at the East manhole yielded a concentration of 4,510 ng/L, which was considerably higher than all samples in the earlier study (Figure 4). Evaluating the actionability of wastewater data for prescription opioids would require an in-depth comparison of prescription rates vs. wastewater concentrations, which was outside the scope of this study.

#### 3.2.4. Stimulants: methamphetamine, amphetamine, cocaine and its metabolites

Other than THC, methamphetamine was the most abundant HRS consumed in Nevada based on the wastewater data in Gerrity et al. (2024). Both methamphetamine and its metabolite amphetamine were detected in 100% of samples in that study, although interpretation of amphetamine data was confounded by its potential for direct consumption through prescription use, and misuse (Teter et al. 2006; Cole & Hussong 2020). In fact, amphetamine for the treatment of ADHD was the 14th most prescribed drug in the U.S. in 2022 (ClinCalc 2025), and studies indicate that misuse of prescription amphetamine is common among TAY (Teter et al. 2006) and potentially linked to misuse of other drugs (Cole & Hussong 2020). Methamphetamine is also prescribed for treatment of ADHD (as Desoxyn) but only in rare cases due to its potential for addiction and abuse.

**Figure 5 |:**
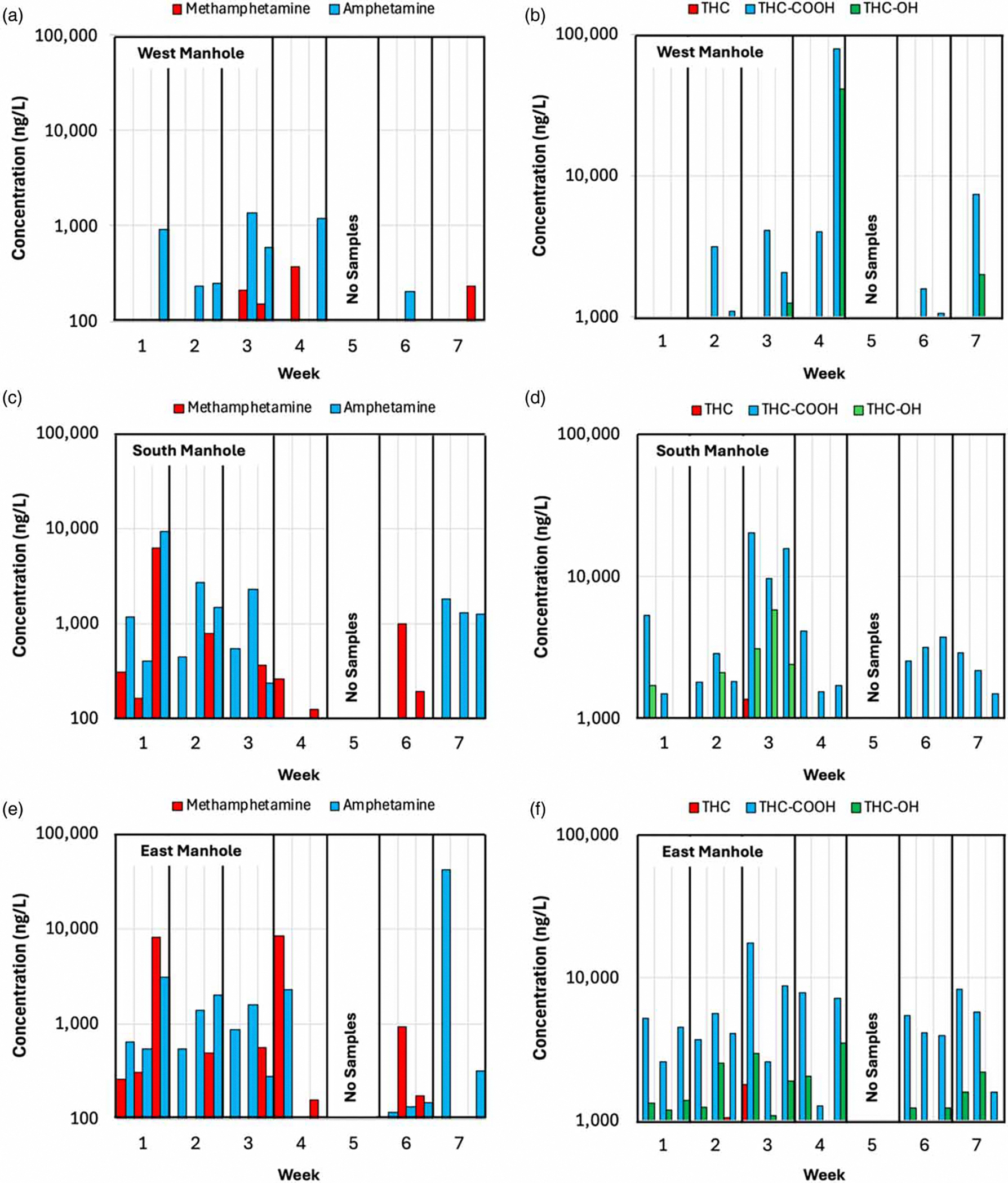
(**Left**) Comparisons of daily methamphetamine and amphetamine concentrations for each sampling week at the (a) West, (c) South, and (e) East manholes. (**Right**) Comparisons of daily THC, THC-COOH, and THC-OH concentrations for each sampling week at the (b) West, (d) South, and (f) East manholes. Each sampling week includes concentrations for the Sunday, Monday, and Tuesday samples.

In the current study, methamphetamine was detected in 22% (West manhole) to 50% (South/East manholes) of all samples. Amphetamine was detected in 39, 67, and 83% of samples from the West, South, and East manholes, respectively (Figure 3), making it the second most frequently detected compound overall. Based on a direct comparison of methamphetamine and amphetamine (Figure 5), detections and concentrations of these compounds appear to be relatively independent, suggesting that amphetamine detections in wastewater are a better indicator of direct amphetamine consumption rather than methamphetamine metabolism. One study at a college in the Pacific Northwest reported spikes in amphetamine and ritalinic acid during finals week (Burgard et al. 2013), but Figure 5 shows that amphetamine detections and concentrations in the current study were generally highest in the weeks immediately following spring break and lowest during finals week and early in the summer session, at which point methamphetamine became more dominant. There was another notable spike in amphetamine use in week 7 (Figure 5), but the reason for this is unclear. As mentioned earlier for prescription opioids, future studies could attempt to correlate wastewater occurrence of amphetamine with prescription data (Schieber et al. 2019) to better differentiate licit vs. illicit use, with the caveat that legally prescribed amphetamine can still be misused.

With the exception of norcocaine, cocaine and its metabolites (see metabolism pathway in Figure S3) were detected in ~100% of samples in the earlier community-scale study (Gerrity et al. 2024). Similar to heroin and its metabolites, the cocaine-related compounds are relatively unstable, but their overall mass balance can still provide a valuable assessment of community-scale cocaine consumption (Gerrity et al. 2024). Relative to the broader sewershed, the wastewater data in the current study suggest low levels of cocaine consumption among contributing individuals, with zero detections of cocaine and norcocaine, singular detections of EME and ecgonine (only at the West manhole), and four detections of BZE scattered across the three manholes (Figure 3). Interestingly, BZE, EME, and ecgonine were all detected in the same Sunday sample from the West manhole (Table S3), providing a strong indication of cocaine consumption. With the exception of that ecgonine detection, concentrations of the cocaine-related compounds were generally lower than the community-scale samples in Gerrity et al. (2024), again highlighting the overall low level of cocaine consumption associated with the campus.

#### 3.2.5. Sedatives: THC, THC-COOH, THC-OH

Considering that marijuana is legal to those aged 21+ in Nevada, it is unsurprising that THC-COOH – the major metabolite of THC – was detected in up to 100% of samples, depending on the sampling location. Consistent with Gerrity et al. (2024), the parent compound THC (log K_OW_ = 7.60; Racamonde et al. 2012) and its other metabolite THC-OH were detected less frequently, partially because the analytical methods were optimized for aqueous-phase compounds rather than compounds likely to sorb to solids. That being said, of all analytes, campus concentrations of the THC-related compounds most closely aligned with those of the corresponding sewershed (Figure 4), presumably due to widespread THC consumption. Although a Tuesday sample from the West manhole yielded the highest THC-COOH and THC-OH concentrations across the entire study (78,800 and 41,200 ng/L, respectively), the West manhole had the lowest detection frequencies of the three locations (50% vs. 94–100 and 22% vs. 33–78%, respectively). Interestingly, April 20th, which is a notable date for cannabis culture, corresponded with a ~10-fold increase in THC-COOH concentrations at the South Manhole across sampling week 3 (Figure 5). Of the three total detections of the parent compound THC in this study, two occurred on that specific date. There was not a corresponding spike in THC-OH concentrations on April 20th, but there was a simultaneous spike in THC-COOH and THC-OH in the aforementioned West manhole sample. Among samples in which both were .MRL, THC-OH concentrations averaged only 35 ± 16% of the corresponding THC-COOH concentrations. As indicators of THC consumption, detections of THC-COOH and THC-OH agreed in only 63% of samples, in part because of lower THC-OH concentrations leading to data censoring.

#### 3.2.6. Psychoactive drugs: MDMA, MDA

MDMA and MDA were <MRL in all samples in the current study. Gerrity et al. (2024) noted that, with the exception of the high-tourism sewersheds (i.e., those including the Las Vegas Strip and Downtown Las Vegas), detection of the recreational psychoactive drug MDMA was sporadic and highly influenced by special events. Moreover, MDA was detected in only a single sample in that study – a sample collected immediately following a fall music festival that also contained MDMA at >1,000 ng/L. Thus, the results for the current study are not entirely surprising. Importantly, sampling at the university campus was suspended during week 5 to divert wastewater monitoring resources for youth and TAY to another music festival known to impact MDMA concentrations in local wastewater (Sims et al. 2024).

### 3.3. Sampling considerations

#### 3.3.1. Grab vs. composite sampling at the south manhole

Composite samples are generally recommended for WBE because they are assumed to be more representative of overall conditions. Particularly in small communities or service areas, grab samples may be more likely to miss critical slugs of wastewater, or even exaggerate average conditions due to non-representative slugs (Gerrity et al. 2022a). Even though the type of sample may yield quantifiable differences in community-scale datasets, multiple studies have demonstrated that grab sampling can be an effective alternative to composite sampling (George et al. 2022; Kmush et al. 2022; Gerrity et al. 2024), particularly when composite sampling is not practical or possible. For reference, Sewershed 4B in Gerrity et al. (2024), which is the corresponding sewershed for the current study, was monitored using grab samples since a composite sampler was not available at that location.

Composite sampling was not possible early in the current study, but the project team later acquired an automated composite sampler, thereby allowing for a direct comparison at the South manhole (Table 1). For that date (May 5th) in week 4, which was during finals week, the only compound detected in the routine grab sample was THC-COOH. The composite sample, however, yielded detection of THC-COOH in addition to acetylmorphine, amphetamine, BZE, methamphetamine, and morphine. Notably, the acetylmorphine detection was the second instance of heroin consumption linked to the South manhole (i.e., the housing complex).

This comparison suggests that composite sampling will sometimes detect additional targets that grab sampling would otherwise miss, particularly in smaller service areas with lower flow rates (George et al. 2022). However, grab sample detections, such as the week 1 detections of heroin and acetylmorphine, can still offer reliable and actionable information for public health response, and grab samples may be the only option when flows are so low that composite sampling is unreliable or impossible from a practical perspective. Alternatively, a recent study demonstrated the efficacy of granular activated carbon (GAC) passive sampling for viruses and bacteria (Ghosh et al. 2026), which should also be effective for HRS and other trace organic compounds. Thus, wastewater monitoring programs must consider the implications of sample type and frequency when collecting manual or automated composites, but practitioners must also account for practical considerations, such as the additional ‘wear and tear’ caused by excessive subsampling (Rocha et al. 2026). Ideal sampling frequencies will ultimately be program- or analyte-specific, and some low prevalence compounds may be best captured by passive sampling. Importantly, negative results from grab samples – or composite samples for that matter – should not necessarily be equated with the definitive absence of consumption in a particular area.

#### 3.3.2. Day of week effects

No occupancy data were requested for any of the buildings included in this study, but there was a visible reduction in occupancy of the academic buildings (i.e., West manhole) on Sundays, when those buildings were closed to the public for the weekend. While Tuesdays yielded the highest detection frequency (14%) across all manholes and target compounds, Sundays and Mondays were just slightly lower at 10%. The day of week effect was more pronounced by location, with the West manhole yielding an overall detection frequency of only 2% on Sundays, consistent with the academic building closures. The West manhole detection frequency increased to ~10% on Mondays and Tuesdays, whereas the combined detection frequencies for the South/East manholes were 14, 11, and 17% on Sundays, Mondays, and Tuesdays, respectively. This suggests that sampling may not be warranted in buildings expected to have low occupancy (i.e., Sundays in the academic buildings), but prioritizing in this manner could still result in critical misses, such as the aforementioned Sunday cocaine signal at the West manhole.

### 3.4. Study limitations

With respect to study design, important limitations include the reliance on grab sampling, except for the one composite sample, and the limited scope of the study at a single, relatively small university campus. However, the grab samples in this study still yielded important and potentially actionable data, suggesting that grab sampling can be a viable alternative when composite sampling is impractical or impossible. A central analytical limitation is method sensitivity for overdose-relevant targets. The direct injection method with 1:10 dilution aimed to increase sample throughput and reduce matrix effects, but this was accomplished at the expense of elevated MRLs (fentanyl and norfentanyl = 50 ng/L, xylazine = 250 ng/L). This is a more conservative approach, increasing confidence in actual detections, but this might also increase false-negative risk when prevalence is low. Another consideration is target scope: the target compound list was robust (24 compounds/metabolites) but could be expanded to include additional overdose-relevant markers (e.g., naloxone), emerging drug classes such as nitazenes (Bade et al. 2025), and adulterants (Erickson et al. 2021). Future work could also pair targeted quantitation with non-target screening to flag emergent compounds (Vogel et al. 2024).

Although not an objective of the current study, back-calculating total and individual consumption estimates based on wastewater concentrations would require corresponding flow data, which was not collected as part of this study. Also, low and variable flow can pose issues for automated composite samplers, providing further justification for grab sampling or passive sampling (Ghosh et al. 2026) in some instances. When grab samples are used, normalization to human-specific markers such as sucralose (Gerrity et al. 2024) and creatinine (Burgard et al. 2013) may be valuable for estimating domestic wastewater inputs and dilution effects (Erickson et al. 2021). Normalization may also allow for more accurate concentration comparisons when service areas differ in size. To minimize analytical burden, sucralose analysis was omitted from this campus-based pilot study. Finally, further validation and/or characterization of wastewater monitoring data, including differentiating illicit vs. licit drug use, could be achieved with corresponding prescription data. However, these datasets can be difficult and costly to acquire.

### 3.5. Implications for public health

This pilot study demonstrates the potential of WBE for real-time situational monitoring of substance use within the on-campus population, offering a timelier signal by weeks to months relative to traditional public health surveillance systems. The findings also serve as a starting point for future public health interventions; the occasional opioid-associated detections in this study may guide future overdose prevention messaging and resource allocation (e.g., naloxone). Other actions informed by these data might include future awareness campaigns and allocation of on-campus wellness resources. Efforts may also consider harm reduction activities like drug take-back events and provision of test strips for fentanyl and xylazine, though there were no wastewater-based detections of fentanyl, norfentanyl, or xylazine in this pilot.

The use of opioid-related wastewater data as an actionable public health surveillance tool is still largely underexplored on U.S. campuses, with few demonstrated examples or success stories. This emerging tool may still be too novel and uncertain for widespread adoption in public health surveillance, highlighting the importance of additional case studies in demonstrating the potential of this tool to fill critical data gaps about substance use on college campuses. Future studies should incorporate operational and financial resources for stakeholder-driven interventions, such as naloxone and fentanyl test strips procurement, sponsored drug take-back events (Bade et al. 2025), and staff time for outreach, education, and training (Le et al. 2025). These findings can be directly translated into evidence-informed public health action on college campuses.

## 4. CONCLUSIONS

Numerous studies and nationwide programs have demonstrated the value of WBE in the context of public health pathogen surveillance. The ongoing opioid epidemic in the U.S. is another potential application of this emerging public health tool, but practical confidence depends on case studies that show how wastewater findings can trigger proportionate, feasible interventions.

A single wastewater sample can provide insight into the health status of tens of thousands of people through examination of diverse targets such as pathogens, opioids, or even markers of chronic disease. This level of efficiency becomes increasingly important in the face of an uncertain funding landscape for public health surveillance and intervention activities (Wildes 2025). Diversifying WBE targets, such as expanding beyond infectious disease targets to incorporate opioids and other HRS, increases the resiliency of WBE infrastructure while extending its ability to protect public health.

This pilot study demonstrated how diverse stakeholders could be engaged in a public health-centric effort to assess HRS consumption among Nevada’s youth and TAY. Results demonstrated the potential to link HRS detections in waste water to upstream locations with high confidence, highlighting an opportunity for public health action within the campus community. Especially when compared to community-wide findings, even non-detects in wastewater samples provide value when they indicate that consumption of a priority target (e.g., fentanyl) is low – if occurring at all. However, before such conclusions are reached, it is important to understand the limitations of a given wastewater sampling scheme and potential impacts on results. For example, reliance on grab sampling rather than composite sampling could miss critical slugs of wastewater that might otherwise indicate fentanyl use/exposure. Nevertheless, this study demonstrates that targeted wastewater monitoring, particularly when complementing community-scale WBE, can be effective in filling critical public health surveillance data gaps.

## Supplementary Material

Supplementary material

## Figures and Tables

**Table 1 |:** Comparison of target compound concentrations in grab vs. composite samples collected on Monday, May 5th (week 4)

Compound	Grab (ng/L)	Composite (ng/L)

**Acetylmorphine**	**<50**	**57**
**Amphetamine**	**<100**	**1,210**
**Benzoylecgonine**	**<50**	**117**
Cocaine	<50	<50
Codeine	<50	<50
EDDP	<100	<100
Ecgonine	<50	<50
EME	<50	<50
Fentanyl	<50	<50
Heroin	<100	<100
Hydrocodone	<100	<100
MDA	<100	<100
MDMA	<100	<100
Methadone	<50	<50
**Methamphetamine**	**<100**	**1,800**
**Morphine**	**<50**	**85**
Norcocaine	<50	<50
Norfentanyl	<50	<50
Oxycodone	<50	<50
THC	<1,000	<1,000
THC-COOH	1,540	1,620
THC-OH	<1,000	<1,000
Tramadol	<50	<50
Xylazine	<250	<250

Bold text indicates compounds for which concentrations differed between the sample types by >10%.

## Data Availability

All relevant data are included in the paper or its Supplementary Information.

## HIGHLIGHTS

- Campus wastewater samples were analyzed for high-risk substances and metabolites.
- Detections involving methamphetamine, amphetamine, and THC were most frequent; fentanyl, norfentanyl, and xylazine were below method reporting limits.
- Campus-based wastewater monitoring builds public health surveillance capacity and can inform future interventions.
- Collaboration and communication with key stakeholders are essential.
