# Supplementary material for: Piloting wastewater-based monitoring on a university campus to inform public health surveillance and response for opioids and other high-risk substances
Source: J Water Health. Author manuscript; Available in PMC 2026 Jul 5. (PMC13333207; doi:10.2166/wh.2026.182)
Supplement: Supplementary material [file NIHMS2172839-supplement-Supplementary_material.pdf]

Supplementary Information

Piloting Wastewater-Based Monitoring on a University Campus to Inform Public Health Surveillance and Response for Opioids and Other High-Risk Substances

Daniel Gerrity[a*+], Casey A. Barber[a+], Rebecca A. Trenholm[a], Andrew Black[a,b], Edwin C. Oh[c], Anil T. Mangla,[d,e] Cassius Lockett,[d] Brett J. Vanderford[a]

[a] Southern Nevada Water Authority, P.O. Box 99954, Las Vegas, NV 89193

[b] University of Wisconsin-Platteville, Department of Civil & Environmental Engineering, Platteville, WI, USA

[c] University of Nevada, Las Vegas, Laboratory of Neurogenetics and Precision Medicine, College of Sciences; Neuroscience Interdisciplinary Ph.D. program; Department of Brain Health; Department of Internal Medicine, Kirk Kerkorian School of Medicine at UNLV, Las Vegas, NV, USA

[d] Southern Nevada Health District, Las Vegas, NV, USA

[e] University of Nevada, Las Vegas, School of Public Health, Las Vegas, NV, USA

*Corresponding Author. Email: daniel.gerrity@snwa.com

[+] These authors contributed equally to this article.

Table of Contents

List of Supplementary Tables

Table S1. High-risk substance and metabolite compound list 6

Table S2. Mass spectrometer source parameters 6

Table S3. Observed target compound concentrations across all samples 11

List of Supplementary Figures

Figure S1. Photo of South manhole isolation of student housing/apartment complex flows 3

Figure S2. Photos and schematic of autosampler 4

Figure S3. Metabolism pathways for heroin and cocaine 7

Figure S4. Project flyer for public outreach 8

Figure S5. Biobot/NIDA wastewater concentrations of fentanyl and norfentanyl 10

List of Supplementary Texts

Text S1. Supplemental analytical methods 5

Figure S1. Photo of South manhole isolation of student housing complex flows.

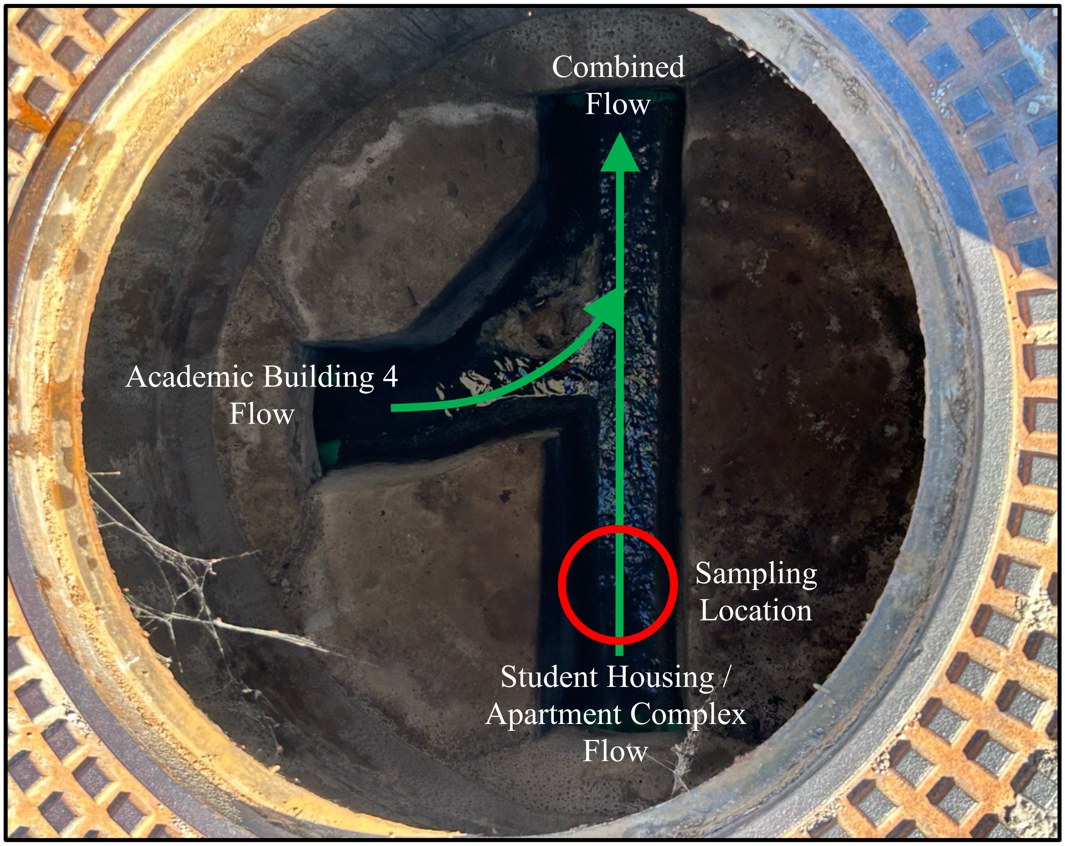

Figure S2. (A) Photo of CA101 autosampler (C.E.C. Analytics, Calgary, AB, Canada). **(B)** Photo of the inside of the CA101 autosampler. **(C)** Photo of the CA101 autosampler suspended in a manhole (not the manhole sampled during study). **(D)** Schematic of the CA101 autosampler and associated dimensions of the suspension and sampling apparatus.

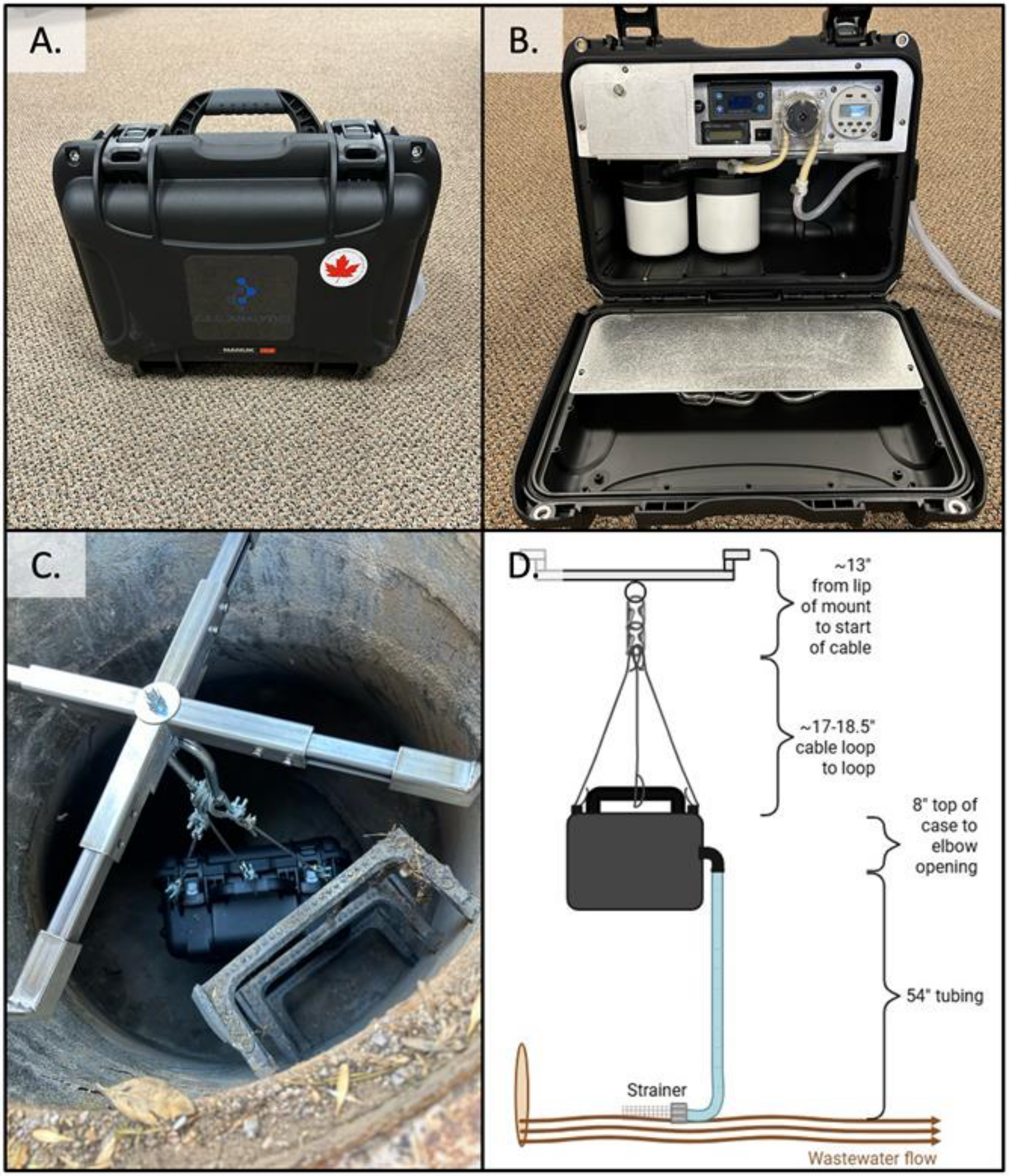

Text S1. Supplemental analytical methods.

The high-risk substances and metabolites listed in **Table S1** were analyzed in each manhole wastewater sample by direct injection liquid chromatography tandem mass spectrometry (LC-MS/MS) after a 1:10 dilution (or greater when necessary) with reagent water. A 100 µL sample loop (sample volume) was used for each injection. Prior to injection, 50 µL of a 50-200 µg/L stock solution of isotopically labeled analogs (Cerilliant, Round Rock, TX, USA) was added to a 10 mL aliquot of each sample. This resulted in a final concentration of 0.25-1.0 µg/L of each isotope, which served as the basis for isotope dilution quantitation. Separation was performed on a 150 × 4.6 mm Raptor Biphenyl column with a 5 µm particle size (Restek, Bellefonte, PA, USA) at room temperature. LC mobile phases consisted of (A) 0.1% formic acid in reagent water and (B) 0.1% formic acid in methanol. The LC flow rate was set at a constant rate of 700 µL/min with a gradient as follows: initial = 5% B, at 0.5 min = 25% B, at 14 min = 100% B, hold until 22 min, followed by a 5 min equilibration at 5% B. The mass spectrometry source parameters are listed in **Table S2**.

A method detection limit (MDL) study was performed using reagent water fortified with the target compounds, each spiked at 5 ng/L, except for THC, THC-OH, and THC-COOH which were spiked at 50 ng/L. MDLs were calculated with the appropriate student's t-value (n=12), and method reporting limits (MRLs) were set at approximately 3-5 times the MDL and adjusted for the dilution factor. Target compounds, MRLs, and multiple reaction monitoring (MRM) transitions are listed in **Table S1**. Calibration curves were made fresh for each analysis by spiking concentrated stock solutions (50/100 µg/L in methanol) accordingly in 10 mL of reagent water, along with isotopes. Calibration ranged from 5-1,250 ng/L, except for THC, THC-OH,

and THC-COOH which ranged from 10-2,500 ng/L. All standards were purchased from Cerilliant, except for ecgonine which was purchased from Restek (Centre County, PA, USA).

Table S1. High-risk substance and metabolite compound list with associated isotopes, method reporting limits (MRLs), and multiple reaction monitoring (MRM) transitions.

Compound	Isotope	MRL (ng/L)	Q1 (m/z)	Q3 (m/z)
6-Acetylmorphine	d6-6-acetylmorphine	50	328	165 (211)
Amphetamine	d8-amphetamine	100	136	91 (119)
Benzoylecgonine	d8-benzoylecgonine	50	290	168 (105, 82.3)
Cocaine	d3-cocaine	50	304	182 (82, 105)
Codeine	d6-codeine	50	300	152 (115)
EDDP	d3-EDDP	50	278	234 (186, 219)
Ecgonine	d3-EME[a]	100	186	100.3 (168)
EME	d3-EME	50	200	82 (182)
Fentanyl	d5-fentanyl	50	337	188 (105)
Heroin	d9-heroin	100	370	165 (268)
Hydrocodone	d6-hydrocodone	50	300	199 (128)
MDA	d5-MDA	100	180	105 (133, 77)
MDMA	d5-MDMA	100	194	163 (135, 77)
Methadone	d9-methadone	50	310	105 (265)
Methamphetamine	d8-methamphetamine	100	150	91 (119)
Morphine	d6-morphine	50	286	152 (165)
Norcocaine	d3-norcocaine	50	290	168 (136)
Norfentanyl	d5-norfentanyl	50	233	84 (150)
Oxycodone	d6-oxycodone	50	316	241 (256)
THC	d3-THC	1,000	315	193 (123)
THC-COOH	d9-THC-COOH	1,000	345	193 (299)
THC-OH	d3-THC-OH	1,000	331	193 (201, 313)
Tramadol	d3-tramadol	50	264	58 (42.2)
Xylazine	d6-xylazine	250	221	164 (90)

( ) – confirmation product ions; Abbreviations: EDDP = 2-ethylidene-1,5-dimethyl-3,3-diphenylpyrrolidine; EME = ecgonine methyl ester; MDA = 3,4-methylenedioxyamphetamine; MDMA = 3,4-methylenedioxymethamphetamine; THC = delta-9-tetrahydrocannabinol; THC-OH = 11-hydroxy-delta-9-tetrahydrocannabinol; THC-COOH = 11-nor-9-carboxy-delta-9-tetrahydrocannabinol; [a]d3-ecgonine is not available so ecgonine quantitation is based on the next closest isotope with respect to structure and retention time, which is d3-EME in this method.

Table S2. Mass spectrometer source parameters in electrospray ionization (ESI) positive mode.

Parameter	Value
Curtain gas (CUR)	20
Collision gas (CAD)	12
Ion spray voltage (IS)	5500
Ion source gas 1 (GS1)	60
Ion source gas 2 (GS2)	50
Temperature (TEM)	550
Entrance potential (EP)	10

Figure S3. Metabolism pathways for heroin and cocaine.

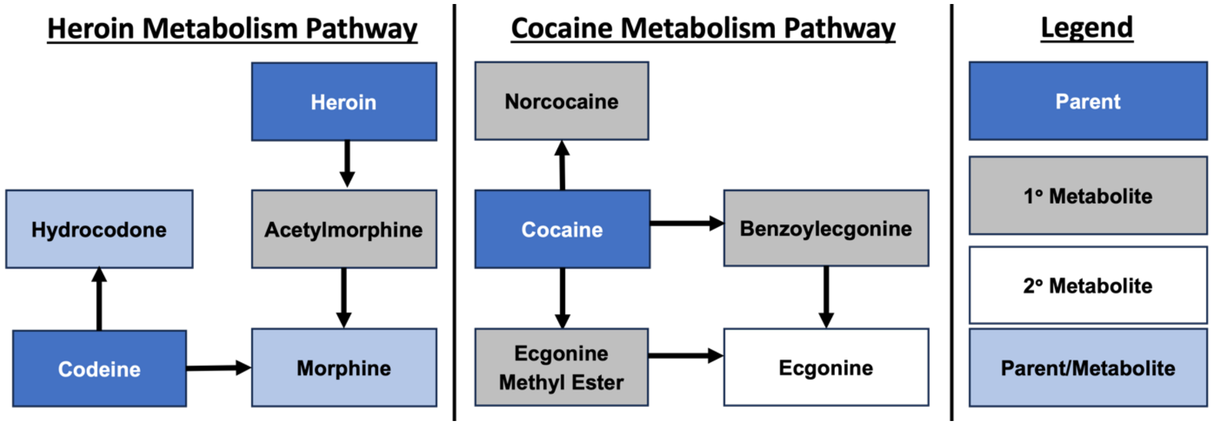

Figure S4. Front of project flyer for public outreach.

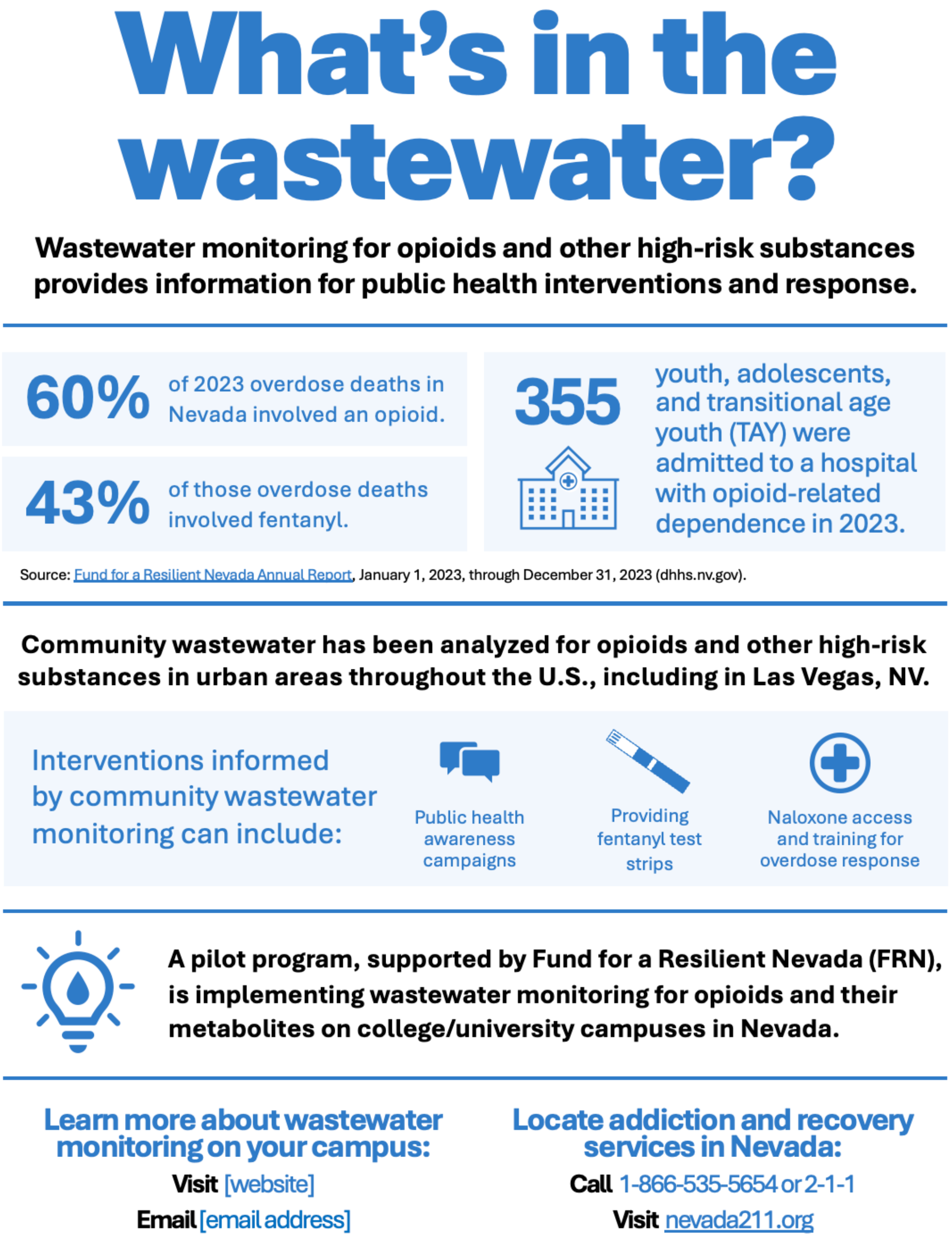

Figure S4 (continued). Back of project flyer for public outreach.

Frequently Asked Questions (FAQs)

What is this project?
This project is implementing a statewide wastewater monitoring program for fentanyl and other opioids (and their metabolites), as well as other sedatives and stimulants.

Where is wastewater monitoring occurring?
Wastewater samples will be collected from one or more utility access holes on or near campus at colleges and universities in Nevada.

Why is this being done at universities?
This program is focused on youth (0-18) and transitional age youth (18-24), which includes college and university students.

What is the timeframe for this project?
During the first phase of this project, wastewater sampling will occur approximately biweekly in late Spring/early Summer of 2025.

What results will be obtained from this project?
Each wastewater sample will be analyzed for the presence and concentration of several drugs and drug metabolites. While some wastewater samples may originate from a specific building's wastewater utility access point, we are not able to identify the specific source location from within that building.

How will the results be shared?
This project will generate reports, summarizing trends in wastewater concentrations of opioids and their metabolites over time, to be shared with project partners including campus administrators, student health and wellness staff, and public health agencies. In the future, results will also be shared on an online dashboard.

What will be done based on the results obtained from this project?
Results may be used to inform public health interventions, including awareness campaigns, trainings, and justification for additional wellness resources, like naloxone supplies or fentanyl test strips.

Results obtained from this project are for public health purposes only. Due to the anonymized nature of wastewater, student, staff, and visitor privacy will remain protected.

How is this project funded?
This project is supported by Fund for a Resilient Nevada (FRN). FRN is administered by the Nevada Department of Health and Human Services (DHHS) to address the impacts, risks, and harms of opioid use in the State of Nevada. FRN is funded by Nevada's allocation of recent opioid litigation recoveries.

How can I learn more about this project?
Visit [website] or email [email address] to learn more.

Figure S5. Influent wastewater concentrations of norfentanyl and fentanyl in Southern Nevada from a national study conducted between October 2023 and August 2024 (one year prior to the campus study). **Source:** Data provided by a collaborating wastewater agency who participated in Biobot's national wastewater monitoring program, which was funded by the National Institute on Drug Abuse (NIDA).

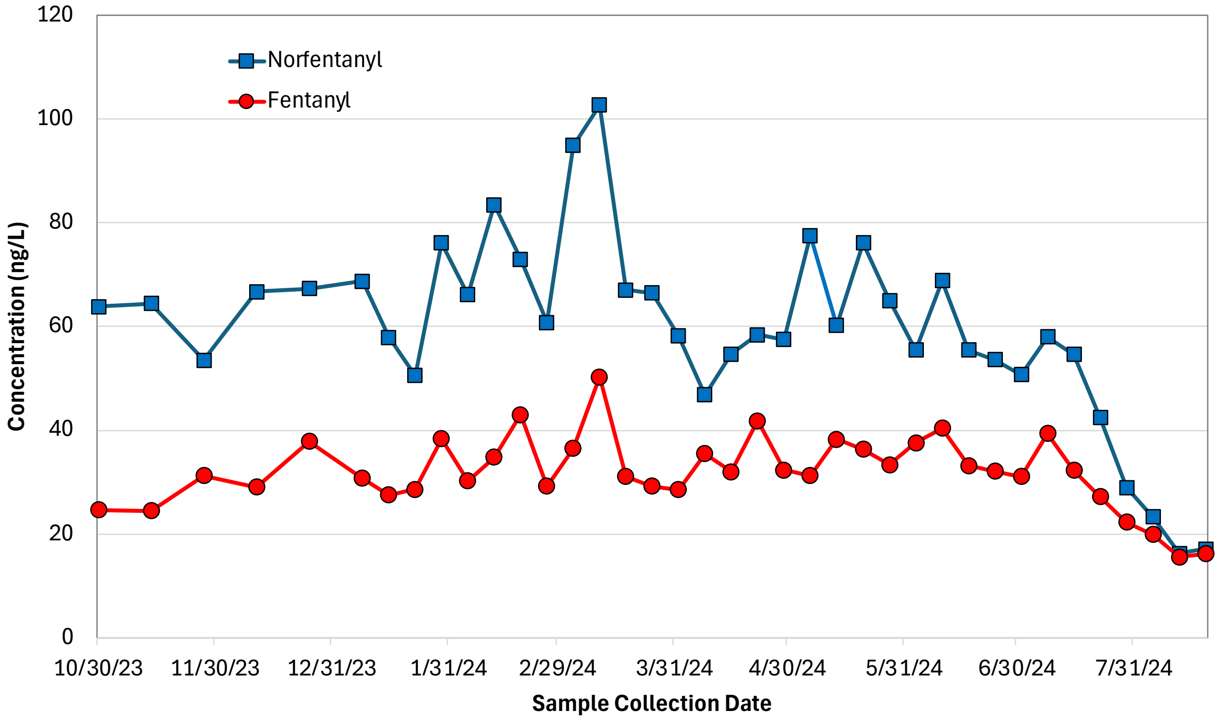

Table S3. Observed concentrations of each analyte in the sewage collected from the West, South, and East manholes. All analytes were non-detect in the weekly field blanks.

Analyte	Week	Day of Week	Date	West	South	East
Fentanyl	1	Sunday	3/23/25	<50.0	<50.0	<50.0
		Monday	3/24/25	<50.0	<50.0	<50.0
		Tuesday	3/25/25	<50.0	<50.0	<50.0
	2	Sunday	4/6/25	<50.0	<50.0	<50.0
		Monday	4/7/25	<50.0	<50.0	<50.0
		Tuesday	4/8/25	<50.0	<50.0	<50.0
	3	Sunday	4/20/25	<50.0	<50.0	<50.0
		Monday	4/21/25	<50.0	<50.0	<50.0
		Tuesday	4/22/25	<50.0	<50.0	<50.0
	4	Sunday	5/4/25	<50.0	<50.0	<50.0
		Monday	5/5/25	<50.0	<50.0	<50.0
		Tuesday	5/6/25	<50.0	<50.0	<50.0
	6	Sunday	6/1/25	<50.0	<50.0	<50.0
		Monday	6/2/25	<50.0	<50.0	<50.0
		Tuesday	6/3/25	<50.0	<50.0	<50.0
	7	Sunday	6/15/25	<50.0	<50.0	<50.0
		Monday	6/16/25	<50.0	<50.0	<50.0
		Tuesday	6/17/25	<50.0	<50.0	<50.0
Norfentanyl	1	Sunday	3/23/25	<50.0	<50.0	<50.0
		Monday	3/24/25	<50.0	<50.0	<50.0
		Tuesday	3/25/25	<50.0	<50.0	<50.0
	2	Sunday	4/6/25	<50.0	<50.0	<50.0
		Monday	4/7/25	<50.0	<50.0	<50.0
		Tuesday	4/8/25	<50.0	<50.0	<50.0
	3	Sunday	4/20/25	<50.0	<50.0	<50.0
		Monday	4/21/25	<50.0	<50.0	<50.0
		Tuesday	4/22/25	<50.0	<50.0	<50.0
	4	Sunday	5/4/25	<50.0	<50.0	<50.0
		Monday	5/5/25	<50.0	<50.0	<50.0
		Tuesday	5/6/25	<50.0	<50.0	<50.0
	6	Sunday	6/1/25	<50.0	<50.0	<50.0
		Monday	6/2/25	<50.0	<50.0	<50.0
		Tuesday	6/3/25	<50.0	<50.0	<50.0
	7	Sunday	6/15/25	<50.0	<50.0	<50.0
		Monday	6/16/25	<50.0	<50.0	<50.0
		Tuesday	6/17/25	<50.0	<50.0	<50.0
Xylazine	1	Sunday	3/23/25	<250	<250	<250
		Monday	3/24/25	<250	<250	<250
		Tuesday	3/25/25	<250	<250	<250
	2	Sunday	4/6/25	<250	<250	<250
		Monday	4/7/25	<250	<250	<250
		Tuesday	4/8/25	<250	<250	<250
	3	Sunday	4/20/25	<250	<250	<250
		Monday	4/21/25	<250	<250	<250
		Tuesday	4/22/25	<250	<250	<250
	4	Sunday	5/4/25	<250	<250	<250
		Monday	5/5/25	<250	<250	<250
		Tuesday	5/6/25	<250	<250	<250
	6	Sunday	6/1/25	<250	<250	<250

Analyte	Week	Day of Week	Date	West	South	East
	6	Monday	6/2/25	<250	<250	<250
		Tuesday	6/3/25	<250	<250	<250
	7	Sunday	6/15/25	<250	<250	<250
		Monday	6/16/25	<250	<250	<250
		Tuesday	6/17/25	<250	<250	<250
Heroin	1	Sunday	3/23/25	<100	<100	<100
		Monday	3/24/25	<100	<100	<100
		Tuesday	3/25/25	<100	482	113
	2	Sunday	4/6/25	<100	<100	<100
		Monday	4/7/25	<100	<100	<100
		Tuesday	4/8/25	<100	<100	<100
	3	Sunday	4/20/25	<100	<100	<100
		Monday	4/21/25	<100	<100	<100
		Tuesday	4/22/25	<100	<100	<100
	4	Sunday	5/4/25	<100	<100	<100
		Monday	5/5/25	<100	<100	<100
		Tuesday	5/6/25	<100	<100	<100
	6	Sunday	6/1/25	<100	<100	<100
		Monday	6/2/25	<100	<100	<100
		Tuesday	6/3/25	<100	<100	<100
	7	Sunday	6/15/25	<100	<100	<100
		Monday	6/16/25	<100	<100	<100
		Tuesday	6/17/25	<100	<100	<100
Acetylmorphine	1	Sunday	3/23/25	<50.0	<50.0	<50.0
		Monday	3/24/25	<50.0	<50.0	<50.0
		Tuesday	3/25/25	<50.0	588	497
	2	Sunday	4/6/25	<50.0	<50.0	<50.0
		Monday	4/7/25	<50.0	<50.0	<50.0
		Tuesday	4/8/25	<50.0	<50.0	<50.0
	3	Sunday	4/20/25	<50.0	<50.0	<50.0
		Monday	4/21/25	<50.0	<50.0	<50.0
		Tuesday	4/22/25	<50.0	<50.0	<50.0
	4	Sunday	5/4/25	<50.0	<50.0	<50.0
		Monday	5/5/25	<50.0	<50.0	<50.0
		Tuesday	5/6/25	<50.0	<50.0	<50.0
	6	Sunday	6/1/25	<50.0	<50.0	<50.0
		Monday	6/2/25	<50.0	<50.0	<50.0
		Tuesday	6/3/25	<50.0	<50.0	<50.0
	7	Sunday	6/15/25	<50.0	<50.0	<50.0
		Monday	6/16/25	<50.0	<50.0	<50.0
		Tuesday	6/17/25	<50.0	<50.0	<50.0
Morphine	1	Sunday	3/23/25	<50.0	<50.0	54
		Monday	3/24/25	<50.0	<50.0	69
		Tuesday	3/25/25	<50.0	353	453
	2	Sunday	4/6/25	<50.0	<50.0	<50.0
		Monday	4/7/25	<50.0	<50.0	<50.0
		Tuesday	4/8/25	<50.0	141	76
	3	Sunday	4/20/25	<50.0	<50.0	<50.0
		Monday	4/21/25	<50.0	<50.0	<50.0
		Tuesday	4/22/25	<50.0	51	73
	4	Sunday	5/4/25	<50.0	<50.0	<50.0
		Monday	5/5/25	<50.0	<50.0	<50.0

Analyte	Week	Day of Week	Date	West	South	East
	4	Tuesday	5/6/25	<50.0	<50.0	<50.0
	6	Sunday	6/1/25	<50.0	<50.0	<50.0
		Monday	6/2/25	<50.0	<50.0	<50.0
		Tuesday	6/3/25	<50.0	<50.0	<50.0
	7	Sunday	6/15/25	<50.0	<50.0	<50.0
		Monday	6/16/25	<50.0	<50.0	<50.0
		Tuesday	6/17/25	<50.0	<50.0	<50.0
Codeine	1	Sunday	3/23/25	<50.0	<50.0	<50.0
		Monday	3/24/25	<50.0	<50.0	<50.0
		Tuesday	3/25/25	<50.0	<50.0	<50.0
	2	Sunday	4/6/25	<50.0	<50.0	<50.0
		Monday	4/7/25	<50.0	<50.0	<50.0
		Tuesday	4/8/25	<50.0	<50.0	<50.0
	3	Sunday	4/20/25	<50.0	<50.0	<50.0
		Monday	4/21/25	<50.0	<50.0	<50.0
		Tuesday	4/22/25	<50.0	<50.0	72
	4	Sunday	5/4/25	<50.0	<50.0	<50.0
		Monday	5/5/25	<50.0	<50.0	<50.0
		Tuesday	5/6/25	<50.0	<50.0	<50.0
	6	Sunday	6/1/25	<50.0	<50.0	<50.0
		Monday	6/2/25	<50.0	<50.0	<50.0
		Tuesday	6/3/25	<50.0	<50.0	<50.0
	7	Sunday	6/15/25	<50.0	<50.0	<50.0
		Monday	6/16/25	<50.0	<50.0	<50.0
		Tuesday	6/17/25	<50.0	<50.0	<50.0
Hydrocodone	1	Sunday	3/23/25	<100	<100	<100
		Monday	3/24/25	<100	<100	<100
		Tuesday	3/25/25	<100	<100	<100
	2	Sunday	4/6/25	<100	<100	<100
		Monday	4/7/25	<100	<100	<100
		Tuesday	4/8/25	<100	<100	<100
	3	Sunday	4/20/25	<100	<100	<100
		Monday	4/21/25	<100	<100	<100
		Tuesday	4/22/25	<100	<100	<100
	4	Sunday	5/4/25	<100	<100	<100
		Monday	5/5/25	<100	<100	<100
		Tuesday	5/6/25	<100	<100	<100
	6	Sunday	6/1/25	<100	<100	<100
		Monday	6/2/25	<100	<100	<100
		Tuesday	6/3/25	<100	<100	<100
	7	Sunday	6/15/25	<100	<100	<100
		Monday	6/16/25	<100	<100	<100
		Tuesday	6/17/25	<100	<100	<100
Oxycodone	1	Sunday	3/23/25	<50.0	<50.0	<50.0
		Monday	3/24/25	<50.0	<50.0	<50.0
		Tuesday	3/25/25	<50.0	<50.0	<50.0
	2	Sunday	4/6/25	<50.0	<50.0	<50.0
		Monday	4/7/25	<50.0	<50.0	<50.0
		Tuesday	4/8/25	<50.0	<50.0	<50.0
	3	Sunday	4/20/25	<50.0	<50.0	<50.0
		Monday	4/21/25	<50.0	<50.0	<50.0
		Tuesday	4/22/25	<50.0	<50.0	<50.0

Analyte	Week	Day of Week	Date	West	South	East
	4	Sunday	5/4/25	<50.0	<50.0	<50.0
		Monday	5/5/25	<50.0	<50.0	<50.0
		Tuesday	5/6/25	<50.0	<50.0	<50.0
	6	Sunday	6/1/25	<50.0	<50.0	<50.0
		Monday	6/2/25	<50.0	<50.0	<50.0
		Tuesday	6/3/25	<50.0	<50.0	<50.0
	7	Sunday	6/15/25	<50.0	<50.0	<50.0
		Monday	6/16/25	<50.0	<50.0	<50.0
		Tuesday	6/17/25	<50.0	<50.0	<50.0
Tramadol	1	Sunday	3/23/25	<50.0	<100	<100
		Monday	3/24/25	<50.0	<100	<100
		Tuesday	3/25/25	<50.0	<100	<100
	2	Sunday	4/6/25	<50.0	<50.0	<50.0
		Monday	4/7/25	<50.0	<50.0	4,510
		Tuesday	4/8/25	<50.0	<50.0	<50.0
	3	Sunday	4/20/25	<50.0	<50.0	<50.0
		Monday	4/21/25	<50.0	<50.0	<50.0
		Tuesday	4/22/25	<50.0	<50.0	<50.0
	4	Sunday	5/4/25	<50.0	<50.0	<50.0
		Monday	5/5/25	<50.0	<50.0	109
		Tuesday	5/6/25	205	<50.0	<50.0
	6	Sunday	6/1/25	<50.0	<50.0	<50.0
		Monday	6/2/25	<50.0	<50.0	<50.0
		Tuesday	6/3/25	<50.0	<50.0	<50.0
	7	Sunday	6/15/25	<50.0	<50.0	<50.0
		Monday	6/16/25	<50.0	<50.0	<50.0
		Tuesday	6/17/25	<50.0	<50.0	<50.0
Methadone	1	Sunday	3/23/25	<50.0	<50.0	<50.0
		Monday	3/24/25	<50.0	<50.0	<50.0
		Tuesday	3/25/25	<50.0	<50.0	<50.0
	2	Sunday	4/6/25	<50.0	<50.0	<50.0
		Monday	4/7/25	<50.0	<50.0	<50.0
		Tuesday	4/8/25	<50.0	<50.0	<50.0
	3	Sunday	4/20/25	<50.0	<50.0	<50.0
		Monday	4/21/25	<50.0	<50.0	<50.0
		Tuesday	4/22/25	<50.0	<50.0	<50.0
	4	Sunday	5/4/25	<50.0	<50.0	<50.0
		Monday	5/5/25	<50.0	<50.0	<50.0
		Tuesday	5/6/25	<50.0	<50.0	<50.0
	6	Sunday	6/1/25	<50.0	<50.0	<50.0
		Monday	6/2/25	<50.0	<50.0	<50.0
		Tuesday	6/3/25	<50.0	<50.0	<50.0
	7	Sunday	6/15/25	<50.0	<50.0	<50.0
		Monday	6/16/25	<50.0	<50.0	<50.0
		Tuesday	6/17/25	<50.0	<50.0	<50.0
EDDP	1	Sunday	3/23/25	<50.0	<50.0	<50.0
		Monday	3/24/25	<50.0	<50.0	<50.0
		Tuesday	3/25/25	<50.0	<50.0	<50.0
	2	Sunday	4/6/25	<50.0	<50.0	<50.0
		Monday	4/7/25	<50.0	<50.0	<50.0
		Tuesday	4/8/25	<50.0	<50.0	<50.0
	3	Sunday	4/20/25	<50.0	<50.0	<50.0

Analyte	Week	Day of Week	Date	West	South	East
	3	Monday	4/21/25	<50.0	<50.0	<50.0
		Tuesday	4/22/25	<50.0	<50.0	<50.0
	4	Sunday	5/4/25	<50.0	<50.0	<50.0
		Monday	5/5/25	<50.0	<50.0	<50.0
		Tuesday	5/6/25	<50.0	<50.0	<50.0
	6	Sunday	6/1/25	<50.0	<50.0	<50.0
		Monday	6/2/25	<50.0	<50.0	<50.0
		Tuesday	6/3/25	<50.0	<50.0	<50.0
	7	Sunday	6/15/25	<50.0	<50.0	<50.0
		Monday	6/16/25	<50.0	<50.0	<50.0
		Tuesday	6/17/25	<50.0	<50.0	<50.0
Methamphetamine	1	Sunday	3/23/25	<100	306	260
		Monday	3/24/25	<100	165	310
		Tuesday	3/25/25	<100	6,260	8,010
	2	Sunday	4/6/25	<100	<100	<100
		Monday	4/7/25	<100	<100	<100
		Tuesday	4/8/25	<100	770	497
	3	Sunday	4/20/25	<100	<100	<100
		Monday	4/21/25	211	<100	<100
		Tuesday	4/22/25	151	367	564
	4	Sunday	5/4/25	<100	256	8,450
		Monday	5/5/25	370	<100	<100
		Tuesday	5/6/25	<100	123	159
	6	Sunday	6/1/25	<100	<100	<100
		Monday	6/2/25	<100	983	930
		Tuesday	6/3/25	<100	190	174
	7	Sunday	6/15/25	<100	<100	<100
		Monday	6/16/25	<100	<100	<100
		Tuesday	6/17/25	235	<100	<100
Amphetamine	1	Sunday	3/23/25	<100	1,160	637
		Monday	3/24/25	<100	400	535
		Tuesday	3/25/25	919	9,370	3,040
	2	Sunday	4/6/25	<100	439	545
		Monday	4/7/25	234	2,720	1,390
		Tuesday	4/8/25	248	1,480	1,960
	3	Sunday	4/20/25	<100	542	868
		Monday	4/21/25	1,380	2,300	1,570
		Tuesday	4/22/25	604	232	273
	4	Sunday	5/4/25	<100	<100	2,310
		Monday	5/5/25	<100	<100	<100
		Tuesday	5/6/25	1,210	<100	<100
	6	Sunday	6/1/25	<100	<100	117
		Monday	6/2/25	204	<100	133
		Tuesday	6/3/25	<100	<100	147
	7	Sunday	6/15/25	<100	1,800	41,700
		Monday	6/16/25	<100	1,270	<1000
		Tuesday	6/17/25	<100	1,240	314
Cocaine	1	Sunday	3/23/25	<50.0	<50.0	<50.0
		Monday	3/24/25	<50.0	<50.0	<50.0
		Tuesday	3/25/25	<50.0	<50.0	<50.0
	2	Sunday	4/6/25	<50.0	<50.0	<50.0
		Monday	4/7/25	<50.0	<50.0	<50.0

Analyte	Week	Day of Week	Date	West	South	East
	2	Tuesday	4/8/25	<50.0	<50.0	<50.0
	3	Sunday	4/20/25	<50.0	<50.0	<50.0
		Monday	4/21/25	<50.0	<50.0	<50.0
		Tuesday	4/22/25	<50.0	<50.0	<50.0
	4	Sunday	5/4/25	<50.0	<50.0	<50.0
		Monday	5/5/25	<50.0	<50.0	<50.0
		Tuesday	5/6/25	<50.0	<50.0	<50.0
	6	Sunday	6/1/25	<50.0	<50.0	<50.0
		Monday	6/2/25	<50.0	<50.0	<50.0
		Tuesday	6/3/25	<50.0	<50.0	<50.0
	7	Sunday	6/15/25	<50.0	<50.0	<50.0
		Monday	6/16/25	<50.0	<50.0	<50.0
		Tuesday	6/17/25	<50.0	<50.0	<50.0
Benzoylecgonine	1	Sunday	3/23/25	<50.0	<50.0	<50.0
		Monday	3/24/25	<50.0	<50.0	<50.0
		Tuesday	3/25/25	<50.0	<50.0	<50.0
	2	Sunday	4/6/25	<50.0	<50.0	<50.0
		Monday	4/7/25	<50.0	<50.0	<50.0
		Tuesday	4/8/25	<50.0	<50.0	<50.0
	3	Sunday	4/20/25	<50.0	76	122
		Monday	4/21/25	<50.0	<50.0	<50.0
		Tuesday	4/22/25	<50.0	<50.0	<50.0
	4	Sunday	5/4/25	<50.0	<50.0	384
		Monday	5/5/25	<50.0	<50.0	<50.0
		Tuesday	5/6/25	<50.0	<50.0	<50.0
	6	Sunday	6/1/25	<50.0	<50.0	<50.0
		Monday	6/2/25	<50.0	<50.0	<50.0
		Tuesday	6/3/25	<50.0	<50.0	<50.0
	7	Sunday	6/15/25	899	<50.0	<50.0
		Monday	6/16/25	<50.0	<50.0	<50.0
		Tuesday	6/17/25	<50.0	<50.0	<50.0
Ecgonine Methyl Ester	1	Sunday	3/23/25	<50.0	<50.0	<50.0
		Monday	3/24/25	<50.0	<50.0	<50.0
		Tuesday	3/25/25	<50.0	<50.0	<50.0
	2	Sunday	4/6/25	<50.0	<50.0	<50.0
		Monday	4/7/25	<50.0	<50.0	<50.0
		Tuesday	4/8/25	<50.0	<50.0	<50.0
	3	Sunday	4/20/25	<50.0	<50.0	<50.0
		Monday	4/21/25	<50.0	<50.0	<50.0
		Tuesday	4/22/25	<50.0	<50.0	<50.0
	4	Sunday	5/4/25	<50.0	<50.0	<50.0
		Monday	5/5/25	<50.0	<50.0	<50.0
		Tuesday	5/6/25	<50.0	<50.0	<50.0
	6	Sunday	6/1/25	<50.0	<50.0	<50.0
		Monday	6/2/25	<50.0	<50.0	<50.0
		Tuesday	6/3/25	<50.0	<50.0	<50.0
	7	Sunday	6/15/25	153	<50.0	<50.0
		Monday	6/16/25	<50.0	<50.0	<50.0
		Tuesday	6/17/25	<50.0	<50.0	<50.0
Ecgonine	1	Sunday	3/23/25	<100	<100	<100
		Monday	3/24/25	<100	<100	<100
		Tuesday	3/25/25	<100	<100	<100

Analyte	Week	Day of Week	Date	West	South	East
	2	Sunday	4/6/25	<100	<100	<100
		Monday	4/7/25	<100	<100	<100
		Tuesday	4/8/25	<100	<100	<100
	3	Sunday	4/20/25	<100	<100	<100
		Monday	4/21/25	<100	<100	<100
		Tuesday	4/22/25	<100	<100	<100
	4	Sunday	5/4/25	<100	<100	<100
		Monday	5/5/25	<100	<100	<100
		Tuesday	5/6/25	<100	<100	<100
	6	Sunday	6/1/25	<100	<100	<100
		Monday	6/2/25	<100	<100	<100
		Tuesday	6/3/25	<100	<100	<100
	7	Sunday	6/15/25	542	<100	<100
		Monday	6/16/25	<100	<100	<100
		Tuesday	6/17/25	<100	<100	<100
Norcocaine	1	Sunday	3/23/25	<50.0	<50.0	<50.0
		Monday	3/24/25	<50.0	<50.0	<50.0
		Tuesday	3/25/25	<50.0	<50.0	<50.0
	2	Sunday	4/6/25	<50.0	<50.0	<50.0
		Monday	4/7/25	<50.0	<50.0	<50.0
		Tuesday	4/8/25	<50.0	<50.0	<50.0
	3	Sunday	4/20/25	<50.0	<50.0	<50.0
		Monday	4/21/25	<50.0	<50.0	<50.0
		Tuesday	4/22/25	<50.0	<50.0	<50.0
	4	Sunday	5/4/25	<50.0	<50.0	<50.0
		Monday	5/5/25	<50.0	<50.0	<50.0
		Tuesday	5/6/25	<50.0	<50.0	<50.0
	6	Sunday	6/1/25	<50.0	<50.0	<50.0
		Monday	6/2/25	<50.0	<50.0	<50.0
		Tuesday	6/3/25	<50.0	<50.0	<50.0
	7	Sunday	6/15/25	<50.0	<50.0	<50.0
		Monday	6/16/25	<50.0	<50.0	<50.0
		Tuesday	6/17/25	<50.0	<50.0	<50.0
THC	1	Sunday	3/23/25	<1000	<1000	<1000
		Monday	3/24/25	<1000	<1000	<1000
		Tuesday	3/25/25	<1000	<1000	<1000
	2	Sunday	4/6/25	<1000	<1000	<1000
		Monday	4/7/25	<1000	<1000	<1000
		Tuesday	4/8/25	<1000	<1000	1,050
	3	Sunday	4/20/25	<1000	1,360	1,780
		Monday	4/21/25	<1000	<1000	<1000
		Tuesday	4/22/25	<1000	<1000	<1000
	4	Sunday	5/4/25	<1000	<1000	<1000
		Monday	5/5/25	<1000	<1000	<1000
		Tuesday	5/6/25	<1000	<1000	<1000
	6	Sunday	6/1/25	<1000	<1000	<1000
		Monday	6/2/25	<1000	<1000	<1000
		Tuesday	6/3/25	<1000	<1000	<1000
	7	Sunday	6/15/25	<1000	<1000	<1000
		Monday	6/16/25	<1000	<1000	<1000
		Tuesday	6/17/25	<1000	<1000	<1000
THC-COOH	1	Sunday	3/23/25	<1000	5,350	5,200

Analyte	Week	Day of Week	Date	West	South	East
	1	Monday	3/24/25	<1000	1,500	2,580
		Tuesday	3/25/25	<1000	<1000	4,510
	2	Sunday	4/6/25	<1000	1,800	3,680
		Monday	4/7/25	3,160	2,870	5,610
		Tuesday	4/8/25	1,100	1,830	4,060
	3	Sunday	4/20/25	<1000	20,300	17,500
		Monday	4/21/25	4,120	9,660	2,570
		Tuesday	4/22/25	2,070	15,800	8,790
	4	Sunday	5/4/25	<1000	4,110	7,830
		Monday	5/5/25	4,030	1,540	1,270
		Tuesday	5/6/25	78,800	1,710	7,170
	6	Sunday	6/1/25	<1000	2,530	5,420
		Monday	6/2/25	1,580	3,180	4,140
		Tuesday	6/3/25	1,060	3,760	3,950
	7	Sunday	6/15/25	<1000	2,890	8,330
		Monday	6/16/25	7,380	2,180	5,780
		Tuesday	6/17/25	<1000	1,490	1,580
THC-OH	1	Sunday	3/23/25	<1000	1,710	1,330
		Monday	3/24/25	<1000	<1000	1,190
		Tuesday	3/25/25	<1000	<1000	1,390
	2	Sunday	4/6/25	<1000	<1000	1,240
		Monday	4/7/25	1,000	2,100	2,530
		Tuesday	4/8/25	<1000	<1000	<1000
	3	Sunday	4/20/25	<1000	3,100	2,960
		Monday	4/21/25	<1000	5,830	1,090
		Tuesday	4/22/25	1,250	2,410	1,890
	4	Sunday	5/4/25	<1000	<1000	2,050
		Monday	5/5/25	<1000	<1000	<1000
		Tuesday	5/6/25	41,200	<1000	3,490
	6	Sunday	6/1/25	<1000	<1000	1,220
		Monday	6/2/25	<1000	<1000	<1000
		Tuesday	6/3/25	<1000	1,000	1,230
	7	Sunday	6/15/25	<1000	<1000	1,590
		Monday	6/16/25	2,000	<1000	2,190
		Tuesday	6/17/25	<1000	<1000	<1000
MDMA	1	Sunday	3/23/25	<100	<100	<100
		Monday	3/24/25	<100	<100	<100
		Tuesday	3/25/25	<100	<100	<100
	2	Sunday	4/6/25	<100	<100	<100
		Monday	4/7/25	<100	<100	<100
		Tuesday	4/8/25	<100	<100	<100
	3	Sunday	4/20/25	<100	<100	<100
		Monday	4/21/25	<100	<100	<100
		Tuesday	4/22/25	<100	<100	<100
	4	Sunday	5/4/25	<100	<100	<100
		Monday	5/5/25	<100	<100	<100
		Tuesday	5/6/25	<100	<100	<100
	6	Sunday	6/1/25	<100	<100	<100
		Monday	6/2/25	<100	<100	<100
		Tuesday	6/3/25	<100	<100	<100
	7	Sunday	6/15/25	<100	<100	<100
		Monday	6/16/25	<100	<100	<100

Analyte	Week	Day of Week	Date	West	South	East
	7	Tuesday	6/17/25	<100	<100	<100
MDA	1	Sunday	3/23/25	<100	<100	<100
		Monday	3/24/25	<100	<100	<100
		Tuesday	3/25/25	<100	<100	<100
	2	Sunday	4/6/25	<100	<100	<100
		Monday	4/7/25	<100	<100	<100
		Tuesday	4/8/25	<100	<100	<100
	3	Sunday	4/20/25	<100	<100	<100
		Monday	4/21/25	<100	<100	<100
		Tuesday	4/22/25	<100	<100	<100
	4	Sunday	5/4/25	<100	<100	<100
		Monday	5/5/25	<100	<100	<100
		Tuesday	5/6/25	<100	<100	<100
	6	Sunday	6/1/25	<100	<100	<100
		Monday	6/2/25	<100	<100	<100
		Tuesday	6/3/25	<100	<100	<100
	7	Sunday	6/15/25	<100	<100	<100
		Monday	6/16/25	<100	<100	<100
		Tuesday	6/17/25	<100	<100	<100
